# Effect of Melatonin Treatment on Storage Quality and Antioxidant System of Postharvest Winter Jujube (*Zizyphus jujube* Mill. cv. Dongzao)

**DOI:** 10.3390/foods14040576

**Published:** 2025-02-10

**Authors:** Hongai Hei, Heyin Tang, Rui Zhao, Gengchen Li, Fei Shi

**Affiliations:** College of Food Science and Engineering, Shanxi Agricultural University, Jinzhong 030801, China; heiheihongai@163.com (H.H.); tangheyin163@163.com (H.T.); rui442753263@163.com (R.Z.);

**Keywords:** melatonin, winter jujube, postharvest, storage quality, antioxidant system

## Abstract

Low-temperature storage is an effective method to extend the shelf life of harvested winter jujube fruit; however, the quality of winter jujube fruit decreases after refrigeration. To enhance the quality of winter jujube with minimum qualitative deterioration during cold storage, this study investigated the effects of different melatonin concentration (100, 150, and 200 µmol/L) treatments on the storage quality of winter jujube (during the white ripening stage) at 0 ± 1 °C (RH, 90 ± 5%) for 90 days. The relative indexes of the fruit quality and the antioxidant system were measured every 15 days. The results showed that different concentrations of melatonin treatments could maintain the fruit’s firmness, retard the fruit’s redness, and inhibit the decrease in weight, total soluble solid (TSS), titratable acidity (TA), and the contents of total phenols, flavonoids, glutathione, and ascorbic acid; they also inhibited the increase in relative conductivity, malondialdehyde (MDA), and hydrogen peroxide (H_2_O_2_) content of jujube fruits and enhanced antioxidant enzyme activities (superoxide dismutase (SOD), catalase (CAT), ascorbate peroxidase (APX), glutathione reductase (GR), and peroxidase (POD)). As a whole, the 200 µmol/L melatonin treatment had the best effect. Therefore, melatonin treatment can maintain the cold-stored quality of postharvest winter jujube by improving its antioxidant capacity.

## 1. Introduction

Winter jujube (*Ziziphus jujuba* Mill.) is widely cultivated in China, and Yuncheng in Shanxi Provence is one of its primary production regions [1]. Its fruit is fresh and crunchy, with a thin and juicy skin. It is rich in nutrients, and it has a high edibility rate of 96.9%. The vitamin C content of jujube is 200–600 mg·100 g^−1^, so it has the reputation of being the “king of all fruits” and the “living vitamin pill” [2]. Fresh jujube fruit also contains amino acids, polyphenols, flavonoids, polysaccharides, triterpenoids, and essential minerals, which have the effect of enhancing immune function, lowering blood pressure, lowering cholesterol, and preventing cardiovascular disease, as well as antioxidant effects, anti-cancer effects, anti-inflammatory effects, and liver protection effects; therefore, jujube fruit is popular with consumers [3,4].

However, fresh winter jujubes are susceptible to serious postharvest quality deterioration, such as rotting, dehydration, tissue softening, and flesh browning, which affects their edible quality and commercial value. Cold storage is an effective method to extend the shelf life of harvested winter jujube fruit; however, the quality of winter jujube fruit decreases after refrigeration. Therefore, several strategies have been explored to improve the physiological quality of fresh jujubes during cold storage. For example, sodium silicate and chitosan treatment effectively reduced the natural decay rate and slowed lesion growth in winter jujube [5], and salicylic acid and 1-methylcyclopropene treatment enhanced the quality of jujube [6]. Ginger-oil-based microencapsulation with improper formatting offered higher nutritional quality for jujube fruit [7], propyl gallate treatment maintained the integrity of jujube skin [8], and CaCl_2_ treatment promoted the improvement of flavonoid metabolism in jujube [2]. Luteolin treatment improved jujube fruit quality through the induction of the phenylpropanoid metabolic pathway [9], and glycine betaine treatment inhibited the decline in quality of winter jujube fruit by regulating energy and antioxidant metabolism. Although these approaches can extend the storage time and maintain the fruit quality of winter jujube to a certain extent, exploring and applying some natural products may have better effects and be better for the environment [10].

Melatonin(N-acetyl-5-methoxytryptamine, MT), a tryptophan derivative naturally occurring in many organisms, is recognized for its potent antioxidant properties [11]. In addition, as a natural and safe preservative, it also exhibits strong antioxidant activity and can respond to biotic and abiotic stresses, ultimately enhancing crop yield and delaying fruit senescence [12]. Recent studies have indicated that melatonin is key in slowing down the postharvest senescence of fruits, boosting antioxidant defense, and preserving their quality after harvest. Melatonin treatment can extend the shelf life of fruits and improve the quality of fruits during storage. Proper melatonin treatment delayed the decrease of firmness of postharvest jujube, inhibited the content of soluble solids and titratable acids, and improved the antioxidant capacity of fruits [13]. A report noted that melatonin treatment inhibited the decline of sucrose while slowing changes in glucose and fructose content, while also increasing the content of phenolic compounds and organic acids, thereby maintaining the flavor and nutritional quality of loquat fruits [14]. In grapes, the rachis browning index, the decay development, the weight loss rate, the berry abscission rate, and the respiration rate were significantly decreased, the accumulation of total phenolics and total flavonoids was promoted, and the reduction of anthocyanins and total soluble solids was delayed [15]. Moreover, exogenous melatonin increased defensive enzyme activities, including superoxide dismutase, peroxidize, catalase, and phenylalanine ammonia-lyase of grapes treated with exogenous melatonin [16]. And, melatonin treatment exerted positive effects on improving antioxidant activity and promoting GABA biosynthesis in yellow-flesh peach fruit [17]. In addition, melatonin treatment maintained the orange quality and enhanced the ROS scavenging capacity by increasing the activity and expressions of catalase, superoxide dismutase, ascorbate peroxidase, and glutathione reductase [18]. These findings suggest that melatonin treatment could serve as an effective method to preserve and regulate postharvest physicochemical characteristics; however, the effects of melatonin on the storage quality of winter jujube fruit are still unclear.

Therefore, in this study, we focused on investigating the effect of different melatonin concentrations on the quality of postharvest jujube fruit and alterations in the antioxidant defense system under low-temperature conditions during storage for 90 days. The findings of this study were intended to provide insights into melatonin as a natural agent to prolong the freshness and quality of winter jujube during cold storage and also offer practical applications in postharvest management and storage technologies for jujube.

## 2. Materials and Methods

### 2.1. Material and Treatments

Winter jujube in the white ripe stage was sourced from a plantation in Linyi (Yuncheng City, Shanxi Province, China). The fruits were promptly transported to the laboratory. Those selected for the trial were uniform in size and free of brown spots, mechanical damage, pests, and disease contamination.

**Figure 1:**
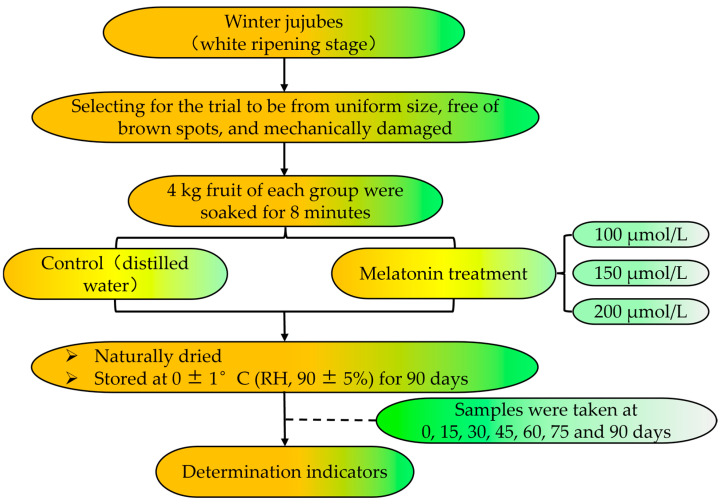
A flow diagram depicting the materials and treatments.

Winter jujubes were split into four groups of 4 kg each. Fruits in each group were soaked in distilled water (control) and 100, 150, and 200 μmol/L of melatonin solution for 8 min, followed by natural air drying. Jujube fruits were put into perforated polyethylene bags (thickness 0.05 mm, size 25 cm × 18 cm) and stored at 0 ± 1 °C (RH, 90 ± 5%) for 90 days, with fruit quality and antioxidant system indexes measured every 15 days (Figure 1). For each group, all measurements were repeated three times.

### 2.2. Chromatic Aberration

Chromatic aberration was determined according to Chang et al. [19]. A colorimeter was used to measure the chromatic aberration (L*, a*, and b*) values of winter jujube peels. Six winter jujubes were randomly selected for each group of samples, and four points were uniformly selected in the equatorial plane of the fruits for measurement.

(1) $$∆E\;=\;\sqrt{{\left(L^{*}-L_{0}\right)}^{2}+{\left(a^{*}-L_{0}\right)}^{2}+{\left(b^{*}-b_{0}\right)}^{2}}$$

### 2.3. Firmness and Weight Loss

According to Chang et al. [20], a texture analyzer (Food Technology Corporation, Sterling, VA, USA) was used to measure the firmness of the jujube fruits. Six jujubes were chosen from each group of samples and placed in a horizontal position on the support table, directly under the P/2 probe. Three parts of the equatorial part of the jujube were selected, and determination was carried out through compression at a distance of 10 mm. The test speed was 60 mm/min, the initial force was 0.5 N, and there was a pause of 2 s between the two compression tests. The post-test speed was 120 mm/min.

The weight of the fruits was measured every 15 days from 20 jujubes in each treatment. The weight loss percentage was calculated based on the initial weight (m_1_) and the weight measured on the corresponding day (m_2_) after storage. The formula is as follows.

(2) $$Weight\;loss(\%)\;=\;\frac{m_{1}-m_{2}}{m_{1}}\;\times \;100$$

### 2.4. TSS and TA

The TSS and TA contents were measured from six winter jujube fruits per treatment. The TSS content was measured using a handheld Brix meter (PAL-1, Atago, Tokyo, Japan), and the results were expressed as (%).

TA was determined based on Wang et al. [21]. The fruit tissue (10 g) was determined through titration with 0.01 mol·L^−1^ of NaOH to pH 8.2 and expressed as citric acid content. The results were recorded as (%).

### 2.5. Relative Conductivity, MDA, H_2_O_2_

Relative conductivity was determined following Ban et al. [22]. Ten winter jujubes were selected randomly and pierced on the equatorial surface of the date with a perforator (10 mm). Then, 15 slices were soaked in 30 mL of deionized water to determine the initial conductivity (L_0_). The samples were then boiled for 10 min and cooled to 20 °C, and the final conductivity (L_1_) was determined.

(3) $$\mathrm{Relative}\;\mathrm{conductivity}(\%)=\frac{L_{0}}{L_{1}}\;\times \;100$$

MDA content was determined following the method of Zhang et al. [23]. The fruit tissue (3 g) was homogenized in 5 mL of 5% TCA and centrifuged at 12,000× *g* for 20 min at 4 °C. Then, 2 mL of supernatant was mixed with 2 mL of 0.67% TBA in 10% TCA and incubated in a boiling water bath for 20 min. After cooling, the absorbance was measured at 450, 532, and 600 nm to calculate MDA. The results were expressed as µmol·kg^−1^.

H_2_O_2_ content was measured using an H_2_O_2_ detection kit (Nanjing Jiancheng, Nanjing, China). The fruit tissues were ground into a homogenate in an ice bath and centrifuged at 10,000× *g* for 10 min at 4 °C, and the supernatant was processed by adding working solution as required. Then, the H_2_O_2_ was measured at 405 nm absorbance, and the results were expressed as mmol·g^−1^ protein.

### 2.6. Total Phenols, Flavonoids, Glutathione, and Ascorbic Acid

According to Wang et al. [24], the jujube fruit tissue was extracted in 1% HCl-methanol solution (4 °C) and protected from light for 20 min, and the extraction solution was measured at 280 nm and 325 nm. Calibration curves with different concentrations of gallic acid and rutin were used to determine the total phenolic and flavonoid content, respectively. The equation y = 0.0018x − 0.0217 demonstrates a linear relationship, with an R^2^ value of 0.9949, where “x” represents the milligram equivalent of gallic acid and ”y” corresponds to the absorbance of the solution at 280 nm. Another equation, y = 0.0009x + 0.0006, demonstrates a linear relationship, with an R^2^ value of 0.9922, where “x” represents the milligram equivalent of rutin and ”y” corresponds to the absorbance of the solution at 325 nm. The results were expressed as U·g^−1^.

Glutathione content was determined based on Tang et al. [25]. A 2.5 mL reaction mixture was prepared to contain supernatant (1 mL), 100 mmol/L of pH 7.7 H_3_PO_4_ solution (1 mL), and 4 mmol/L of DTNB (0.5 mL), and then it was measured at 412 nm. A standard curve was generated with different concentrations of reduced glutathione standard solution. With the equation y = 0.0035x + 0.0002 and an R^2^ value of 0.9994, “x” referred to the glutathione concentration, and “y” represented the absorbance value of the solution at 412 nm. Results are expressed as μmol·g^−1^.

Ascorbic acid content was measured according to Sang et al. [1]. The fruit tissue (4.5 g) was homogenated with 100 mL of 2% oxalic acid and centrifuged at 12,000× *g* for 10 min at 4 °C. We then pipetted 1 mL of the supernatant into a conical flask and titrated it with calibrated 2,6-dichlorophenolindophenol, recording the volume of 2,6-dichlorophenolindophenol solution consumed. We replaced the filtrate with oxalic acid solution as the control. The results were expressed in mg·100 g^−1^.

### 2.7. Antioxidant-System-Related Enzyme Activities

SOD, CAT, APX, and GR activities were determined using an enzyme activity kit (Beijing Solarbio Science & Technology, Beijing, China). Frozen date tissue was homogenized in an ice bath, and appropriate working solutions were added. The activities of SOD, CAT, APX, and GR were measured under the absorbance of 450 nm, 405 nm, 290 nm, and 340 nm, respectively. POD activity was measured using a guaiacol colorimetric assay [25]. We added 3 mL of 25 mM guaiacol solution, 0.5 mL of enzyme extract, and 200 μL of 0.5 mol/L H_2_O_2_ solution, and the absorbance values were measured at 420 nm at one-minute intervals for six consecutive times. It is defined as the increase in absorbance per minute. The results were expressed as U·g^−1^.

### 2.8. Statistical Analysis

All experiments were conducted in triplicate. Statistical analysis and correlation analysis were performed using SPSS v. 20.0 (Armonk, NY, USA). The data were analyzed through one-way analysis of variance, and the date standard errors (±SE, *n* = 3) were calculated with Microsoft Excel. Mean separation was determined using Duncan’s multiple range test at the 5% level. The correlation was analyzed through Pearson correlation analysis. And, figures were prepared using Origin 2021 (Northampton, MA, USA).

## 3. Results

### 3.1. Effects on Fruit Color

**Figure 2:**
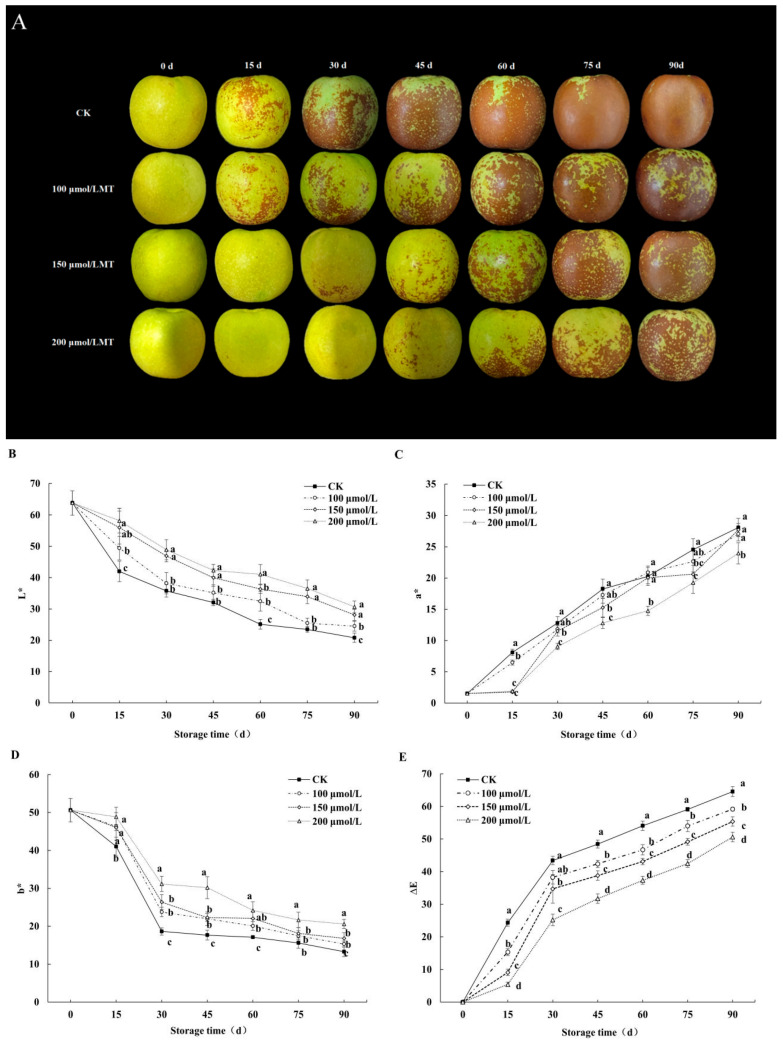
Effects of melatonin treatments on color change (**A**), L* (**B**), a* (**C**), b* (**D**), and ∆E (**E**) of winter jujube during cold storage. The SE is represented by vertical bars, and (a–d) differ significantly (*p* < 0.05).

The results presented in Figure 2A show that the redness occurred in different degrees in melatonin-treated and control fruit peels during cold storage. The pericarp of fruit that underwent 100, 150, and 200 μmol/L melatonin treatment turned red to different degrees from the 15th, 30th, and 45th days of storage, respectively, and the degree of change was lower than that of the control. With regard to fruit color change, the 200 μmol/L melatonin treatment exhibited the most effective results.

The L*, a*, b*, and ∆E values are used to evaluate the variation in color of the jujube fruit’s skin. The L* value indicates the degree of brightness, and the a* and b* values reflect the color shifts along red–green and yellow–blue, respectively. The L* value (Figure 2B) and the b* (Figure 2D) values decreased continuously, while the a* (Figure 2C) and ΔE (Figure 2E) values increased during cold storage. The L* and b* values of the melatonin treatments were significantly higher than those of the control (*p* < 0.05). The a* and ΔE values of the melatonin treatments were significantly lower than those of the control. On the 15th day, the b* values of the control and the 100 μmol/L melatonin treatment increased significantly, but there were no significant changes in the 150 μmol/L and 200 μmol/L melatonin treatments. On the 60th day, the L* values of the 200, 150, and 100 μmol/L melatonin treatments were 38.84%, 30.91%, and 29.24% higher than those of the control, respectively. Among all treatments, the 200 μmol/L melatonin treatment consistently exhibited significantly higher b* values compared to the control and other treatments. On the 30th day, the b* value of the 200 μmol/L melatonin treatment was 67.24% higher than that of the control. Furthermore, on the 60th day, the b* value of the 200 μmol/L melatonin treatment was 31.18% and 18.19% higher than those of the 100 μmol/L and 150 μmol/L melatonin treatments. The ΔE value represents overall color differences. On the 90th day, the ΔE value of the control was 64.54%, which was 21.55%, 14.20%, and 8.34% higher than the values of the 200 μmol/L, 150 μmol/L, and 100 μmol/L melatonin treatments. Among these, the 200 μmol/L melatonin treatment exhibited the lowest ΔE value, indicating the most effective preservation of the original color during storage.

### 3.2. Effects on Fruits’ Firmness and Weight Loss

**Figure 3:**
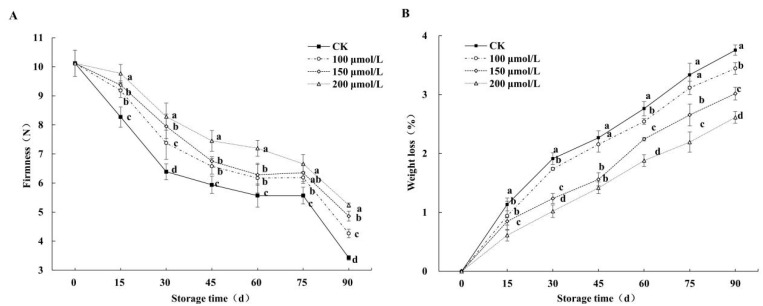
Effects of melatonin treatments on firmness (**A**) and weight loss (**B**) of winter jujube during cold storage. The SE is represented by vertical bars, and (a–d) differ significantly (*p* < 0.05).

The firmness of fruits subjected to melatonin treatments and the control gradually decreased during storage. As shown in Figure 3A, the firmness of the control was markedly lower than that of the fruits that underwent melatonin treatments at the same storage time point. Meanwhile, the firmness of the fruits subjected to 100 μmol/L of melatonin and 150 μmol/L of melatonin showed little difference, while 200 μmol/L of melatonin led to notably higher firmness than the other treatments. On the 15th day, fruit firmness showed the greatest decrease in the control, while the 200 μmol/L melatonin treatment demonstrated a markedly smaller decrease than the other treatments (*p* < 0.05). By the end of storage, the firmness of the control declined to the lowest level, as it was only 3.43 N; however, the firmness of the fruit that underwent the 200 μmol/L melatonin treatment was maintained at 5.24 N, which was 34.64%, 29.55%, and 19.71% higher than values of the control and the 100 and 150 μmol/L melatonin treatments, respectively. In Figure 3B, the fruit weight loss of the control and the fruits that underwent melatonin treatments show a gradually increasing trend during storage, and the weight loss of the control group was the highest, while that of the 200 μmol/L melatonin treatment group was the lowest. At the end of storage, the weight loss of the 100, 150, and 200 μmol/L melatonin treatment groups was 3.45%, 3.02%, and 2.61% lower than that of the control, respectively. The results showed that the melatonin treatments could significantly delay the decline in firmness and reduce the weight loss of the postharvest jujube fruit, with 200 μmol/L of melatonin exhibiting the best effect.

### 3.3. Effects on Fruits’ TSS and TA

**Figure 4:**
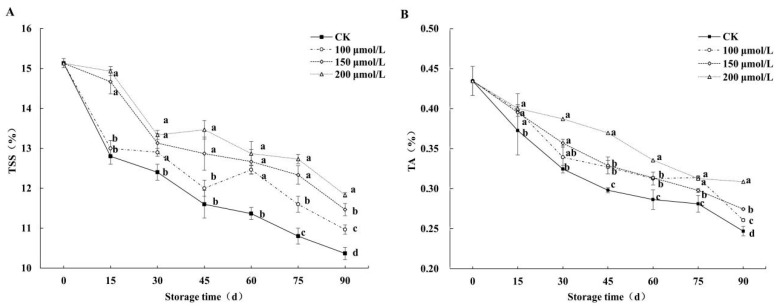
Effects of melatonin treatments on TSS (**A**) and TA (**B**) of winter jujube during cold storage. The SE is represented by vertical bars, and (a–d) differ significantly (*p* < 0.05).

The fruits’ TSS content (Figure 4A) following melatonin treatment was higher than that of the control at the same storage time point. On the 15th day, the TSS contents of the control were 12.8%, while those of the fruits that underwent the 100, 150, and 200 μmol/L melatonin treatments were 13.0%, 14.67%, and 14.93%, respectively. On the 90th day, the TSS content of the 200, 150, and 100 μmol/L melatonin treatment groups was 12.39%, 7.32%, and 3.10%, which was significantly higher (*p* < 0.05) than that of the control. In Figure 4B, the TA contents of the fruits that underwent melatonin treatments were markedly higher than that of the control during storage. There was less of a difference between the 150 μmol/L and 100 μmol/L melatonin treatment groups between 15 and 60 days of storage. On the 90th day, the TA content of the 200 μmol/L, 150 μmol/L, and 100 μmol/L melatonin treatment groups was 20.05%, 10.09%, and 5.34% higher than that of the control. The results indicate that different concentrations of melatonin treatment could affect the content of TSS and TA of jujube during storage, with the 200 μmol/L melatonin treatment exhibiting the best effect.

### 3.4. Effects on Fruits’ Relative Conductivity, MDA, and H_2_O_2_

**Figure 5:**
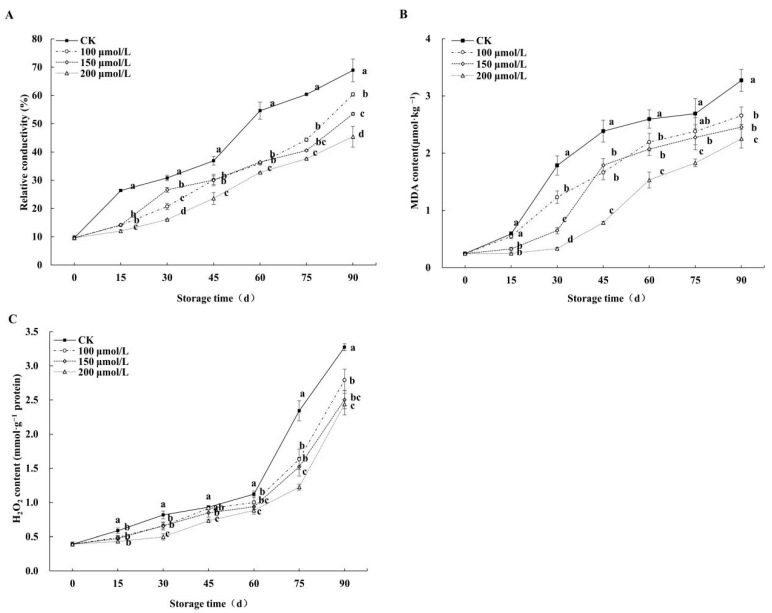
Effects of melatonin treatments on relative conductivity (**A**), MDA (**B**), and H_2_O_2_ (**C**) of winter jujube during cold storage. The SE is represented by vertical bars, and (a–d) differ significantly (*p* < 0.05).

The relative conductivity (Figure 5A) of fruits subjected to melatonin treatments was significantly lower than that of the control, and the relative conductivity of the control group was the highest, while that of the 200 μmol/L melatonin treatment group was the lowest. On the 15th day, the relative conductivity of the control group increased by 63.37% compared to the value at the beginning of storage, while there was no significant change in the melatonin-treated fruit. On the 90th day, the relative conductivity of the fruit subjected to 200, 150, and 100 μmol/L melatonin treatments was 34.11%, 22.41%, and 12.42% lower than that of the control.

The MDA content (Figure 5B) of all groups showed an increasing trend during cold storage, while the MDA content of the melatonin treatment groups was lower than that of the control. On the 30th day, the MDA of the 200 μmol/L melatonin treatment group was only 0.33 µmol·kg^−1^, which was 88.01%, 66.39%, and 16.70% lower than that of the control and the 100 and 150 μmol/L melatonin treatment groups, respectively. The results show that melatonin treatment could inhibit the increase in MDA content in jujube fruits during storage, among which the 200 μmol/L melatonin treatment had the best effect.

The H_2_O_2_ content (Figure 5C) of jujube fruits in the control and the melatonin treatment groups was relatively stable before 60 days of storage. On the 60th day, the H_2_O_2_ content in the control increased sharply. Compared with the control, the trend of H_2_O_2_ content in the melatonin treatment groups was relatively smooth. There was no significant difference between the 150 μmol/L and 100 μmol/L melatonin treatment groups, and the H_2_O_2_ content of fruit that underwent the 200 μmol/L melatonin treatment was at a lower level. By the end of storage, compared with the control, the H_2_O_2_ content of the 100 μmol/L, 150 μmol/L, and 200 μmol/L melatonin treatment groups decreased by 14.69%, 23.52%, and 25.47%, respectively.

### 3.5. Effects on Total Phenols, Flavonoids, Glutathione, and Ascorbic Acid of Fruits

**Figure 6:**
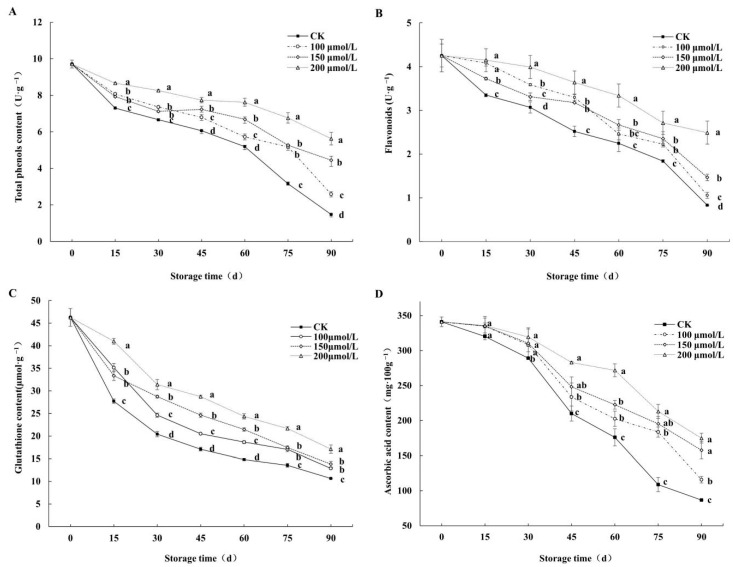
Effects of melatonin treatments on total phenols (**A**), flavonoids (**B**), glutathione (**C**), and ascorbic acid (**D**) of winter jujube during cold storage. The SE is represented by vertical bars, and (a–d) differ significantly (*p* < 0.05).

The content of total phenolics (Figure 6A) and flavonoids (Figure 6B) in each treatment followed an overall decreasing trend during storage. On the 15th day, the total phenolic content of the fruits subjected to the 200 μmol/L, 150 μmol/L, and 100 μmol/L melatonin treatments was 8.66 U·g^−1^, 7.93 U·g^−1^, and 8.07 U·g^−1^, while that of the control was 7.29 U·g^−1^, which was significantly lower (*p* < 0.05) than that of the MT treatment groups. On the 45th day, the total phenolic content of the melatonin treatment groups was significantly higher than that of the control group (*p* < 0.05). On the 90th day, the total phenolic content of the control dropped to 1.48 U·g^−1^; meanwhile, the content of total phenolics of the 200 μmol/L, 150 μmol/L, and 100 μmol/L melatonin treatment groups was 5.63 U·g^−1^, 4.46 U·g^−1^, and 2.58 U·g^−1^, respectively, which was significantly higher than that of the control (*p* < 0.05). In addition, on the 15th day, the flavonoid content of the 200 μmol/L and 150 μmol/L melatonin treatment groups showed little difference, but it was significantly higher than that of the 100 μmol/L melatonin treatment group and the control. During the 45–75 days of storage, there was no significant difference between the 100 μmol/L and 150 μmol/L melatonin treatment groups. At the end of storage, the flavonoid contents of the 100 μmol/L, 150 μmol/L, and 200 μmol/L melatonin treatment groups were 1.06 U·g^−1^, 1.47 U·g^−1^, and 2.49 U·g^−1^, respectively, which were 1.27, 1.77, and 3.01 times higher than those of the control. Therefore, melatonin treatment could significantly maintain the total phenolic content and the flavonoid content of postharvest jujube fruits, and the 200 μmol/L melatonin treatment had the best effect.

The glutathione (Figure 6C) and ascorbic acid (Figure 6D) content of the control was lower than that of the fruit subjected to melatonin treatment during storage. In Figure 6C, on the 15th day, the content of glutathione in the control was 27.70 μmol·g^−1^; however, the content of glutathione in the 200 μmol/L, 150 μmol/L, and 100 μmol/L melatonin treatment groups was 40.99 μmol·g^−1^, 33.35 μmol·g^−1^, and 35.15 μmol·g^−1^. On the 90th day, the glutathione content of the 200, 150, and 100 μmol/L melatonin treatment groups was 17.11 μmol·g^−1^, 13.81 μmol·g^−1^, and 2.89 μmol·g^−1^, respectively, which were increased by 37.83%, 22.98%, and 17.47%, respectively, compared to that of the control group. In Figure 6D, the ascorbic acid content was 341.01 mg·100 g^−1^ at the beginning of fruit storage. On the 45th day, the ascorbic acid content of the control declined to 210.04 mg·100 g^−1^, which was lower than that of the fruit subjected to 200 μmol/L, 150 μmol/L, and 100 μmol/L melatonin treatments by 25.78%, 18.15%, and 11.23%. By the end of storage, the ascorbic acid content of the 200 μmol/L, 150 μmol/L, and 100 μmol/L melatonin treatment groups was 174.85 mg·100 g^−1^, 157.68 mg·100 g^−1^, and 115.81 mg·100 g^−1^, whereas that of the control fruit was only 86.78 mg·100 g^−1^. As a whole, different concentrations of melatonin treatment could effectively delay the decline of glutathione and ascorbic acid content in jujube fruit, with the 200 μmol/L melatonin treatment exhibiting the greatest effect.

### 3.6. Effects on Antioxidant System Enzyme Activities of Fruits

**Figure 7:**
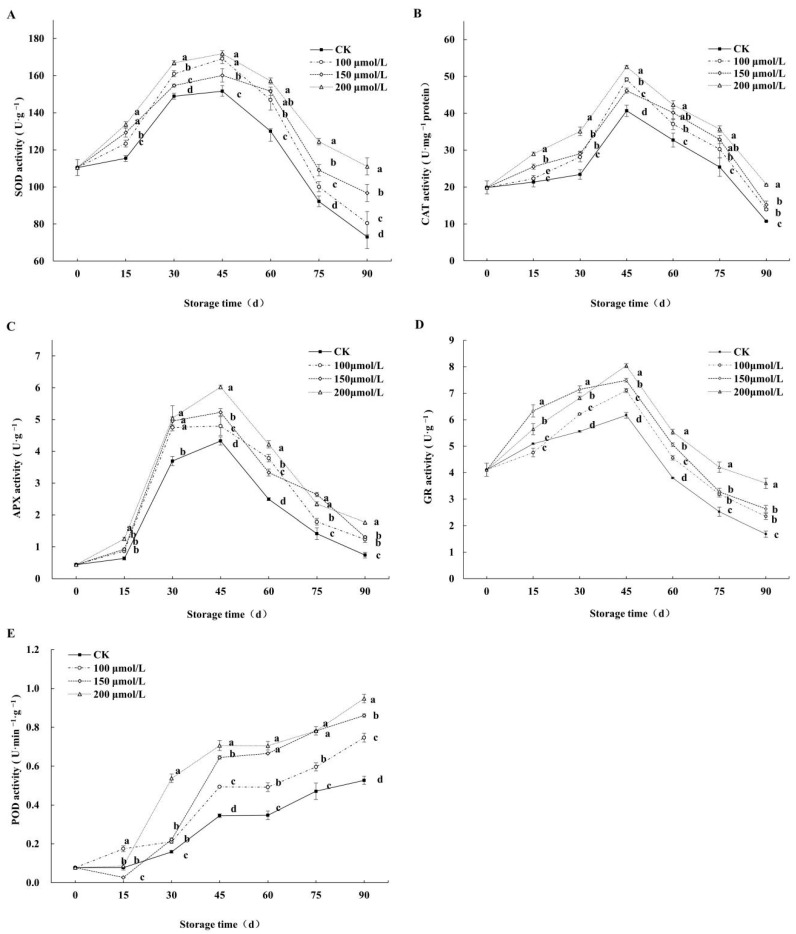
Effects of melatonin treatments on antioxidant system enzyme activities of SOD (**A**), CAT (**B**), APX (**C**), GR (**D**), and POD (**E**) of winter jujube during cold storage. The SE is represented by vertical bars, and (a–d) differ significantly (*p* < 0.05).

The activities of SOD (Figure 7A), CAT (Figure 7B), APX (Figure 7C), and GR (Figure 7D) in the control and melatonin treatment groups followed a pattern of initial increase followed by a subsequent decrease during the fruit storage period. On the 30th day, SOD activity (Figure 7A) in the 200, 150, and 100 μmol/L melatonin treatment groups was markedly higher than that of the control, with increases of 12.08%, 3.87%, and 8.1%, respectively. On the 90th day, SOD activity in the 200, 150, and 100 μmol/L melatonin treatment groups was 1.52, 1.33, and 1.10 times higher than that of the control, respectively. On the 45th day, CAT activity (Figure 7B) peaked, and it was 1.21, 1.13, and 1.29 times higher in the fruits subjected to the 200, 150, and 100 μmol/L melatonin treatments compared to the control. On the 90th day, CAT activity in melatonin treatment groups was higher than in the control by 48.17%, 23.01%, and 29.50%, respectively (*p* < 0.05). Between 15 and 30 days of storage, APX activity (Figure 7C) increased rapidly and peaked at 45 days. From 45 to 90 days, APX activity gradually decreased in both the melatonin treatment groups and the control, with the melatonin treatments maintaining higher levels than the control. On the 90th day, APX activity in the 200, 150, and 100 μmol/L melatonin treatment groups was 1.67, 1.77, and 2.39 times higher than that of the control, respectively. On day 45th, GR activity (Figure 7D) in the 100, 150, and 200 μmol/L melatonin treatment groups was 15.18%, 21.47%, and 30.39% higher than that of the control, respectively. On the 90th day, GR activity in the 200 μmol/L melatonin treatment group was 2.14, 1.53, and 1.37 times greater than in the 150 μmol/L and 100 μmol/L melatonin treatment groups and the control group, respectively. POD activity in the melatonin treatments (Figure 7E) increased rapidly from 15 to 45 days of storage and remained elevated thereafter. By the 45th day, significant differences were observed between the melatonin treatments and the control. On the 90th day, POD activity in the melatonin treatment groups (200, 150, and 100 μmol/L) was 0.44%, 0.39%, and 0.29% higher than that of the control group. Overall, melatonin treatment could significantly increase the activities of SOD, CAT, APX, GR, and POD and improve the antioxidant capacity of postharvest winter jujube fruits.

### 3.7. Correlation Analysis

**Figure 8:**
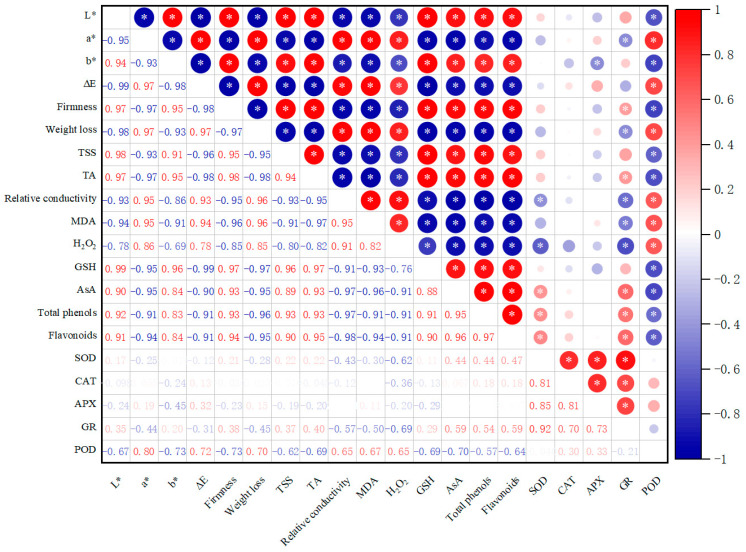
The relevance matrix of appearance (color), firmness, weight loss, and antioxidant system indexes of winter jujube treated with melatonin for 90 days. Red indicates a positive correlation, and blue indicates a negative correlation. Correlation levels are significant at * *p* < 0.05.

As shown in Figure 8, the correlation analysis of the quality indices, the antioxidant-system-related indices, and the enzyme activities throughout the storage of winter jujube fruits revealed significant correlations between firmness, weight loss, color difference, TSS content, TA content, relative electrical conductivity, MDA content, H_2_O_2_ content, and antioxidants, with large correlation coefficients (*p* < 0.05). Significant positive correlations (*p* < 0.05) were observed between firmness, L* value, b* value, TSS, TA, total phenolic content, flavonoids, glutathione, ascorbic acid content, weight loss, a* value, ∆E value, relative conductivity, MDA, and H_2_O_2_ content.

Significant positive correlations were found between the activities of CAT, SOD, GR, and APX, suggesting that these enzymes collaborate to strengthen the antioxidant defense system in jujube fruits during storage. The increased activities of these enzymes likely contribute to better protection against oxidative damage. On the other hand, negative correlations between POD activity and CAT, SOD, GR, and APX activities imply that POD might operate through a different mechanism or respond to distinct oxidative stress cues, indicating a complex interaction within the fruit’s overall antioxidant response during storage.

## 4. Discussion

Fruit changes during storage are closely linked to variations in firmness and weight loss [26,27]. Shang et al. reported that 0.05 mmol·L^−1^ of melatonin treatment effectively extended the storage time to 42 days of blueberry fruit; compared to the control, the firmness of the fruit that underwent melatonin treatment was significantly increased, weight loss was reduced, and the rate of water loss and decay was retarded during this period [28]. Similarly, Carrión-Antolí et al. demonstrated that melatonin treatment could significantly reduce weight loss in two varieties of sweet cherry during storage at 2 °C for 21 days [29]. In our study, melatonin treatment decreased weight loss and delayed the softening of the postharvest jujube fruit during cold storage.

Chromatic aberration serves as a crucial indicator of fruit ripeness and sensory quality, which directly affects its shelf life [30]. Our results showed that melatonin could slow down pigment deposition in winter jujube and delay jujube skin redness. In the correlation analysis, the L* and b* values exhibited a strong positive correlation with firmness, TSS, and TA while negatively correlating with weight loss rate, a*, and ∆E. TSS and TA are the main intrinsic qualities that influence the flavor of the fruit; they are associated with water evaporation during storage and used to evaluate the nutritional value of winter jujube [31]. The results of this paper demonstrated that melatonin effectively slowed the reduction in TSS and TA content. Liu et al. reported that treatment with 0.5 mmol of melatonin effectively delayed changes in mango ripening characteristics, such as fruit firmness, flesh color, β-carotene content, TSS, and TA [32]. The study has reported that melatonin treatment slowed down the jujube’s color change and loss of firmness in dates while preserving TSS and TA levels [33]. These results align with the findings of this paper.

Ascorbic acid is both a vital nutrient and an antioxidant [34]. Glutathione and ascorbic acid can directly scavenge free radicals and eliminate reactive oxygen species, increase the antioxidant capacity of fruit cells, and protect total phenolics and flavonoids from degradation [4]. This study’s results demonstrated that melatonin effectively reduced relative conductivity and MDA content, minimized H_2_O_2_ accumulation, and enhanced the levels of flavonoids, total phenols, glutathione, and ascorbic acid. Xie et al. reported that melatonin treatment notably boosted the levels of total phenolics, flavonoids, and anthocyanins in litchi [35]. Excessive H_2_O_2_ accumulation results in lipid peroxidation, which damages cell membrane integrity. Lipid peroxidation is considered a key factor contributing to postharvest fruit senescence [36]. This process increases the permeability of the cell membrane, leading to elevated relative conductivity, which in turn promotes the softening and ripening of the fruit. MDA, a harmful byproduct of lipid peroxidation in cell membranes, serves as an indicator of membrane damage [37]. There was a report that melatonin treatment enhanced ascorbic acid levels while reducing the accumulation of H_2_O_2_ and MDA, and the relative membrane permeability in sweet cherries [38]. Similarly, Gao et al. found that melatonin significantly reduced the concentrations of H_2_O_2_ and MDA in peaches [39]. Additionally, Zhang et al. demonstrated that melatonin treatment inhibited pericarp browning and the production of reactive oxygen species, such as O^2−^, H_2_O_2_, and MDA, in litchi fruit [40].

Many previous studies have shown that antioxidant-system-related enzymes play a crucial role in scavenging reactive ROS, thereby reducing ROS-induced damage to fruit cells and delaying fruit ripening and senescence during storage. This has been demonstrated in studies on bananas, longans, dragon fruits, and chilies [41,42,43,44]. Zhang et al. reported that treatments with 1-methylcyclopropene and salicylic acid significantly increased the activities of SOD, CAT, APX, GR, and POD in postharvest winter jujubes during the 0–20-day storage period [6]. These findings suggest that these enzymes may be activated during the ripening process of jujubes, enhancing the fruit’s antioxidant capacity, reducing ROS accumulation, minimizing oxidative damage to cells, and maintaining better fruit quality. Similarly, we found that the activities of SOD, CAT, APX, GR, and POD in melatonin-treated jujube fruits were consistently higher compared to the control; therefore, melatonin treatment significantly enhanced the activities of antioxidant enzymes, effectively delaying postharvest senescence of winter jujubes.

## 5. Conclusions

In summary, the winter jujube fruit’s firmness, weight loss rate, L*, a*, and b* value TSS, and TA were related to the total phenolic, flavonoid, glutathione, and ascorbic acid content and the relative conductivity, MDA, and H_2_O_2_ content. The fruit’s quality was improved, the color change and weight loss were decreased after different concentrations of melatonin treatments, and the effect of 200 μmol/L of melatonin treatment was the best. This may be due to decreasing the relative conductivity, MDA, and H_2_O_2_ content and thus increasing the contents of antioxidants (total phenolic, flavonoid, glutathione, ascorbic acid) and the activities of SOD, CAT, APX, GR, and POD in the antioxidant system. These findings revealed that melatonin treatment could be an effective postharvest method for improving the quality of cold-stored fruits; however, the molecular mechanism needs to be further explored. 

## Data Availability

The original contributions presented in the study are included in the article, further inquiries can be directed to the corresponding author.
